# Acute upper airway obstruction due to retropharyngeal hematoma in a dog with *Anaplasma* species: a case study

**DOI:** 10.1186/s12917-015-0574-7

**Published:** 2015-10-09

**Authors:** Verónica Vieitez, María Martín-Cuervo, Víctor López-Ramis, Luis Javier Ezquerra

**Affiliations:** Veterinary Teaching Hospital, University of Extremadura, Avda, Universidad s/n, Cáceres, 10003 Spain

**Keywords:** Canine, Bleeding disorders, Acute airway obstruction, Retropharyngeal hematoma

## Abstract

**Background:**

Retropharyngeal hematoma is a rare condition that is difficult to diagnose and may progress rapidly to airway obstruction. The authors report the first known case of acute upper airway obstruction resulting from retropharyngeal hematoma in a dog. Documented causes in human medicine have included coagulopathic states, trauma, infection, parathyroid adenoma rupture, and foreign body ingestion. Vague symptoms in humans such as sore throat, shortness of breath, dysphonia, dysphagia, and neck swelling may precede lethal airway obstruction.

**Case presentation:**

The authors report a case of an 18-month-old, intact female water spaniel with thrombocytopenia that developed a massive retropharyngeal hematoma and symptoms of airway compromise. The dog required tracheal intubation followed by surgical tracheostomy. Lateral cervical radiography and magnetic resonance imaging of the neck was consistent with a retropharyngeal hematoma compromising the airway. The retropharyngeal hematoma was managed conservatively.

**Conclusion:**

Retropharyngeal hematoma should be considered in patients presenting with abrupt respiratory distress. Magnetic resonance imaging allowed specific diagnosis of a rare condition that is otherwise difficult to diagnose.

## Background

Retropharyngeal hematoma (RH) is a rare condition that can rapidly cause airway obstruction in people [1, 2]. The condition develops when blood collects in the retropharyngeal space, which extends from the base of the skull cranially to the level of the tracheal bifurcation caudally and therefore, provides a route for communication between the neck and the chest [3]. The retropharyngeal space itself contains lymph nodes, fat, and lymphatic channels, but no great vessels or organs [4].

The clinical presentations of RH differ depending on the size of the RH and its rate of growth. RH is characterized by the clinical triad of tracheal and esophageal compression, ventral tracheal displacement, and the subsequent appearance of subcutaneous bruising in the anterior neck and upper thorax [5]. Sore throat, shortness of breath, dysphagia and odynophagia, either alone or in various combinations, may be the initial symptoms. Bruising, tenderness and/or swelling of the neck can sometimes make the diagnosis more obvious [6]. An expanding hematoma in this area may affect the airway at different levels as a result of mass effect. Because of anatomical barriers, the bulk of the hematoma could compress the nasopharynx, oropharynx, hypopharynx, or esophagus; therefore, the obstruction could be at the level of the upper airway, cervical trachea, or intrathoracic trachea. This knowledge is significant, as a standard tracheostomy may not resolve the airway obstruction.

Multiple etiological factors could contribute to RH formation and, if no cause is identified, the condition is characterized as a spontaneous RH. Etiologies of RH include infection, cervical spine trauma, great vessel trauma, violent head movements, iatrogenic injury associated with cardiac catheterization or cerebral angiography, parathyroid adenoma hemorrhage, or foreign body ingestion [1]. Anticoagulation states [7–12] or hemorrhagic diathesis predispose to the development of RH in humans [1]. Precipitating factors such as episodes of coughing, sneezing, straining and vomiting indirectly contribute to RH by increasing venous pressure and causing ruptures in the venous system. RH must be differentiated from abscess, arterial aneurysm, malformation or prominence of the vertebral bodies, and adenoiditis [13].

In human medicine, patients with RH secondary to coagulopathy (e.g., hemophilia, hemorrhagic diathesis, or anticoagulant therapy) present with fewer symptoms and, once the diagnosis is made, intubation or tracheotomy is more difficult [1]. In some patients, 12–48 h of bleeding may precede complaints of respiratory embarrassment; however, an early, seemingly indolent course can suddenly be complicated by airway obstruction. Death can occur following rapid development of respiratory distress from sublingual, retropharyngeal, and parapharyngeal hemorrhages [1]. In the case of RH in patients on therapeutic anti-coagulant therapy, 33 % of patients experienced precipitous airway compromise leading to surgical airway management, and the mortality rate was 10 % [12]. However, in spontaneous RH, there is a reported mortality rate of up to 20 % [14]. In human patients hospitalized for cervical injury, 60 % of RH cases result from the injury but airway obstruction caused directly by the RH occurs rarely, in only 1.2 % of patients [15, 16].

Because a patient’s history can be nonspecific, a high index of suspicion is required to diagnose RH. To aid the diagnosis, lateral cervical radiography can be used to measure abnormal thickness of the retropharyngeal tissue. In veterinary medicine, as a rough estimate, the normal distance between the dorsal edge of the cricoid cartilage and the ventral aspect of C2 is approximately equal to the length of C3 on a properly positioned lateral radiograph [17]. The authors discuss the first known case of retropharyngeal hematoma in a dog that tested positive for *Anaplasma* species.

## Case presentation

An 18-month-old, intact female water spaniel (16 kg) was referred to the Veterinary Teaching Hospital, University of Extremadura, after experiencing weakness, neck pain and progressive paralysis in all limbs that led to lateral recumbency for 7 days. There were no other remarkable findings on physical examination. Complete blood count and serum biochemistry profiles revealed leukocytosis (white blood cell count: 31.5 × 10^9^/L; reference range, 5.9–16. 6 × 10^9^/L); elevated alanine aminotransferase (377 IU/L; reference range, 15–52 IU/L) and alkaline phosphatase (317 IU/L; reference range, 20–70 IU/L); and thrombocytopenia (109 × 10^9^/L; reference range, 117–460 × 10^9^/L). No coagulation testing was performed.

Based on the history and examination findings, possible differential diagnoses included meningitis, polyradiculoneuritis, polymyositis, and discospondylitis. We anesthetized the dog for a cisternal puncture and cerebrospinal fluid (CSF) tap and encountered no complications during the procedure. Analysis of the cerebrospinal fluid showed elevated protein (15 g/L; reference range, < 0.25 g/L) and neutrophilic pleocytosis (white blood cell count, 5.0 × 10^9^/L; reference range < 5 × 10^6^/L; 69 % neutrophils, 39 % lymphocytes). Based on these results, and pending CSF culture, there was a suspicion of bacterial meningitis and the dog was started on antibiotic therapy (sulfamethoxazole-trimethoprim,^1^ 15 mg/kg PO q 12 h and clindamycin,^2^ 10 mg/kg PO q 12 h).

**Fig. 1:**
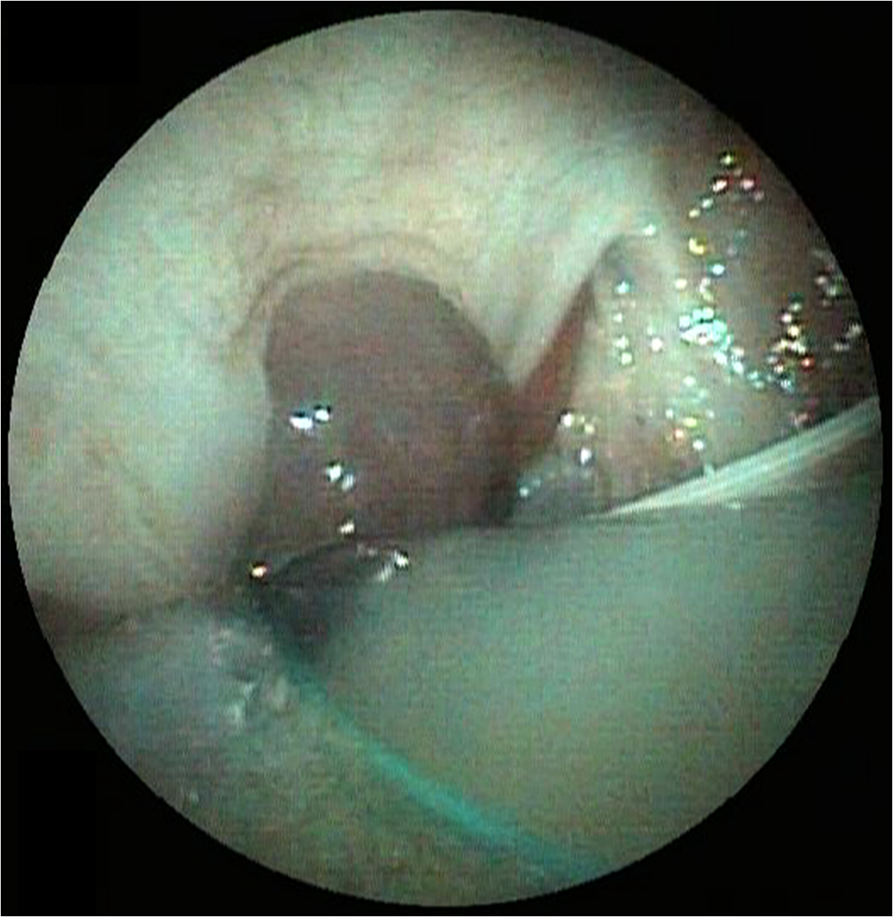
Pharyngoscopy image demonstrating a soft, bulging, violaceous mass involving lateral and dorsal pharyngeal walls

**Fig. 2:**
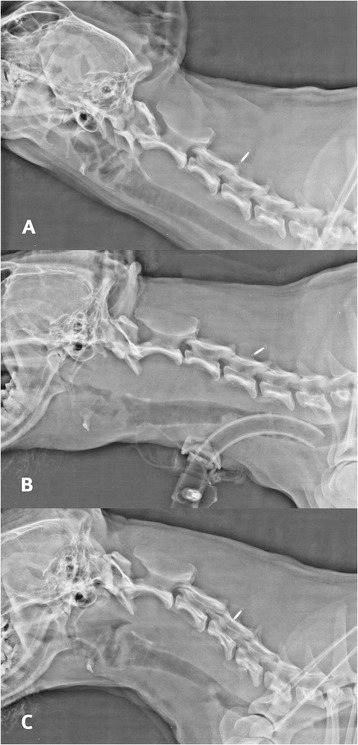
Lateral cervical spine radiographs showing normal (**a**) and increased thickness of the prevertebral soft tissue (**b**, **c**). **a** before anesthesia; **b** during the event; **c** four days after tracheostomy

**Fig. 3:**
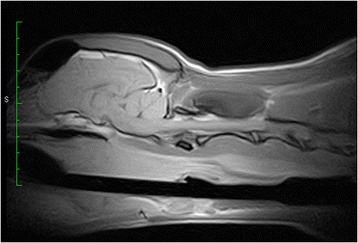
Sagittal T1 magnetic resonance image showing a high signal prevertebral collection spreading from the nasopharynx to C4

Five hours after extubation, the dog developed stridor and evidence of upper airway obstruction that worsened rapidly. The dog was agitated, tachypneic and cyanotic on room air and was immediately transferred to the operating room to secure the airway. Intubation was attempted via direct visualization with laryngoscopy, but this was difficult because of the size of the hematoma. The epiglottis was not visualized and the correct position of the endotracheal tube was confirmed using capnography. On examination of the oral cavity and oropharynx, there was an ecchymotic swelling of the soft palate. Laryngoscopy confirmed extensive hematoma; pharyngoscopy demonstrated a soft, bulging, violaceous mass involving the lateral and dorsal pharyngeal walls (Fig. 1). A lateral cervical radiograph revealed marked prevertebral soft tissue swelling (Fig. 2). The dog was then transferred to the magnetic resonance imaging (MRI) unit under general anesthesia, and urgent MRI^3^ of the neck was performed (Fig. 3). MRI revealed a retropharyngeal mass with features consistent with hemorrhage; the trachea was outlined only by the endotracheal tube and the mass was causing dorsal compression of the airway.

The severe extent of the upper airway obstruction necessitated surgical tracheostomy, which was performed uneventfully, and aminocaproic acid^4^ (20 mg/kg IV), vitamin K^5^ (2 mg/kg IV), and ethamsylate^6^ (12.5 mg/kg IV) were administered. Once the dog was stabilized, blood tests were repeated. Hemoglobin was 109 g/L from 123 g/L, platelet count was 36 × 10^9^/L from 109 × 10^9^/L, and clotting study results were in the normal range (thrombin time: 6.4 s, reference range: 6–9 s; partial thromboplastin time: 5.1 s, reference range: 5.1–7.9 s; prothrombin time: 8.7 s, reference range: 8.6–12.9 s). Despite thrombocytopenia that was just above the range to cause bleeding, buccal mucosal bleeding time was normal (2 min; reference range, 1.7–4.4 min); platelet aggregometry was not performed to assess platelet function. *Anaplasma* and *Ehrlichia* blood antigen detection (4DX^7^) was performed, and the dog tested positive for *Anaplasma spp.*; therefore, prednisone^8^ (2 mg/kg IV q 12 h) and doxycycline^9^ (10 mg/kg PO q 24 h) were initiated as treatment for rickettsial meningitis. Over the next 24 h, the platelet count increased to 153 × 10^9^/L and the hemoglobin concentration stabilized at 109 g/L. The tracheostomy tube was removed on the fourth day and normalization of the prevertebral soft tissue was noted on a lateral cervical spine radiograph (Fig. 2). Based on the clinical and radiological findings, a diagnosis of RH was made. Following negative CSF culture, antibiotic therapy was reduced to doxycycline for one month. Rehabilitation therapy was instituted and in 7 days the dog was able to rise and take a few steps alone. The neurological signs improved with treatment and resolved over the next several months resulting in complete and uneventful recovery.

In this case, a flexible laryngoscope confirmed the diagnosis. The role of flexible pharyngoscopy cannot be overemphasized. Even a small bulging of the posterior wall of the pharynx should alert clinicians and guide the clinical strategy towards a high suspicion of a retropharyngeal space-occupying lesion [18]. Computed tomography is also important to confirm the diagnosis and to assess the extent of the hematoma and its relation to structures in the neck [6]. MRI offers several advantages over computed tomography regarding multiplanar anatomic display and superior soft tissue contrast, allowing more specific diagnoses to be made [19]. MRI not only better depicts the extent of retropharyngeal lesions but, most importantly, is able to identify acute and subacute blood products, thereby affecting both diagnosis and management. MRI is sensitive to blood products in different stages of evolution because of their paramagnetic signal properties, which change over time depending on the dominant component (e.g., acute deoxyhemoglobin, subacute intra- or extracellular methemoglobin, and chronic hemicromes).

Spontaneous bleeding generally does not occur until platelet counts fall below 20–50 × 10^9^/L unless a concomitant bleeding disorder exists, in which case it can be a life-threatening event. Thrombocytopenia does not always require intervention, but it certainly warrants monitoring and attention to prevent bleeding episodes [20]. In this case, the dog responded well to treatment with doxycycline and platelet transfusion was not necessary.

In human medicine, most authors report conservative treatment for successful management of RH, and the condition usually resolves within several weeks [6]. Treatment depends on the size, location, and clinical evolution of the patient. In small hematomas, treatment tends to be conservative, and spontaneous resolution occurs [21]. Immediate opening of the hematoma for aspiration appears to increase the risk of infection, which often extends to the mediastinum. If the hematoma is large and re-absorption unlikely, or if the hematoma has extended rapidly, surgical evacuation and controlling the source of bleeding is indicated once the patient has been stabilized [13]. Late complications include a non-resolving hematoma or abscess formation.

There is little consensus in the literature regarding the use of steroids or prophylactic antibiotics in patients with RH [2] and the use of systemic steroids is controversial. Hemorrhage in the upper airway dissects along multiple fascial planes, and this incites an inflammatory reaction that is limited by the use of steroids. Although not of proven benefit, systemic steroids have been used in numerous case reports of upper airway hematomas in people [22, 23].

## Conclusions

The authors report the first known veterinary case of an RH in a dog. RH should be considered a rare differential for dogs with underlying coagulopathy that develop upper airway compromise.

## Abbreviations

RH: Retropharyngeal hematoma
MRI: Magnetic resonance imaging

## Footnotes

1 Sulfamethoxazole-trimethoprim: Septrin tablets, 800/160 mg, Alcala Farma Laboratories, SL Ctra. M-300, Km 29,920 Alcalá de Henares. 28802 Madrid, Spain

2 Clindamycin: Dalacin tablets 150 mg, Pfizer, SL. Avda. De Europa 20 B, Parque Empresarial La Moraleja. 28108 Alcobendas, Madrid, Spain

3 Vet-MR Grande, Esaote España SA. Sant Just Desvern, Barcelona, Spain

4 Aminocaproic acid: Caproamin Fides, injectable, 4 g, Rottapharm Laboratories, SL, Avda. Diagonal, 67–69, 08019, Barcelona, Spain

5 Vitamin K: Konakion, injectable 10 mg/ml, Roche Farma Laboratories, SA, C/Eucalipto 33, 28016 Madrid, Spain

6 Ethamsylate: Hemo 141, 125 mg/ml, Esteve SA. Avda. Mare de Déu de Montserrat, 221.08041 Barcelona, Spain

7 SNAP 4DX; IDEXX Laboratories, Westbrook, ME, USA

8 Prednisone: Dacortin tablets, 30 mg, Merck, SL. Maria de Molina, 40. 28006. Madrid, Spain

9 Doxycycline: Doxiclat tablets, 100 mg, Pierre Fabre Ibérica, SA. C/Ramón Trias Fargas 7–11. 08005 Barcelona, Spain
